# Adjuvant potency of Astragaloside VII embedded cholesterol nanoparticles for H3N2 influenza vaccine

**DOI:** 10.3906/biy-2003-49

**Published:** 2020-10-13

**Authors:** Rükan GENÇ, Nilgün YAKUBOĞULLARI, Ayşe NALBANTSOY, Fethiye ÇÖVEN, Erdal BEDİR

**Affiliations:** 1 Department of Chemical Engineering, Faculty of Engineering, Mersin Turkey; 2 Department of Bioengineering, Faculty of Engineering, İzmir Institute of Technology, İzmir Turkey; 3 Department of Bioengineering, Faculty of Engineering, Ege University, İzmir Turkey; 4 Veterinary Control and Research Institute, İzmir Turkey

**Keywords:** Immunomodulatory activity, nanocarriers, cholesterol, triterpenoid saponins, Astragaloside VII, influenza

## Abstract

Adjuvants are substances that increase the immune response to a given antigen. In the development of novel vaccine adjuvants/systems, saponins are one of the most attractive molecules due to their altered immunomodulatory activities. In this study, we tried to develop PEG (polyethylene glycol)/cholesterol-based lipid nanoparticles (LNPs) to deliver the Astragaloside VII (AST-VII) and potentiate adjuvant properties of AST-VII for the influenza vaccine. In the formation of PEG/cholesterol/AST-VII-based LNPs (PEG300: Chol-AST-VII LNPs), 3 different primary solvents (acetone, ethanol, and chloroform) were evaluated, employing their effects on hydrodynamic particle size, distribution, surface chemistry, and colloidal stability. Prepared nanoparticles were simply admixtured with inactivated influenza antigen (H3N2) and applied to PMA (phorbol 12-myristate 13-acetate)-ionomycin treated human whole blood to evaluate their cytokine release profile. PEG300: Chol-AST-VII LNPs (80.2 ± 7.7 nm) were obtained using chloroform as a desolvation agent. Co-treatment of PMA-ionomycin with AST-VII and PEG300: Chol-AST-VII LNPs significantly increased the levels of IL-2 and IFN-g, compared to PMA-ionomycin alone. In the presence of H3N2, AST-VII was able to augment IL-17A, while PEG300: Chol-AST-VII LNPs stimulated the production of IFN-g. Hemolysis was only observed in PEG300: Chol-AST-VII LNPs (250 μg/mL) treatment. AST-VII and AST-VII-integrated LNPs could be used as efficacious adjuvants for an inactivated H3N2 vaccine in vitro, and cytokine response through Th1/Th17 route was reported.

## 1. Introduction

Adjuvants are chemical/biological molecules that increase the efficacy of vaccines by augmenting the immunogenicity of weaker immunogens and thereby reduce the amount of antigen and frequency of immunization required for protective immunity (Shi et al., 2019; Wang and Xu, 2020). There are many types of vaccines with adjuvants under clinical trials or in the market that mostly serve as oil-in-water emulsions and phospholipid formulations (Ilyinskii et al., 2014). One of the significant challenges in developing an effective adjuvant carrying formulation that combines the adjuvant and vaccine is finding the optimal carrier system that potentiates immune responses and can ensure the delivery of the cargo with higher bioavailability (Malyala and Singh, 2009).

Influenza viruses (IVs) are globally important human respiratory pathogens belonging to the orthomyxovirus family. These single-stranded and helically-shaped RNA viruses are divided into 3 main types (A, B, and C) in terms of nuclear material. IVs were first named in the “Hong Kong” pandemic (1968) and, since then, they have been responsible for several particularly significant epidemics throughout history. Seasonal IVs frequently evolve, and new strains quickly replace the older. This entails the need for a periodical update and reassessment of vaccine effectiveness (Harding and Heaton, 2018).

The development of novel adjuvant systems or the improvement of adjuvants to promote cellular and humoral immune response plays a crucial role in providing more robust protection against IVs. Lipid-based carriers are an essential class of adjuvant carriers that can encapsulate both hydrophilic and hydrophobic cargo (Sardan et al., 2013). Cholesterol is one of the well-known liposomal components used mostly to increase membrane flexibility and permeability. Lipid nanoparticles (LNPs) containing mRNA coding protein antigens have been used to induce immune response and provide protection against influenza and Zika viruses in mice (Shirai et al., 2020). For this reason, LNPs can be used as an effective delivery vehicle for peptide/protein antigens and adjuvant in the vaccine development sector. LNPs have been developed to deliver immunostimulatory agents such as CpG [Toll Like Receptor (TLR-9) agonist] (Shirota and Klinman, 2014), MPL (monophosphoryl lipid A, TLR-4 agonist) (Kedmi et al., 2010), and small-molecule immune potentiators targeting TLR-7 (Wu et al., 2014). In LNP production, saponins are also used due to their immunostimulatory properties. Saponins are secondary metabolites derived from plant or marine organisms and have a wide range of commercial and industrial applications in the pharmaceutical and food industries. They are characterized by their aglycones as triterpenoid or steroidal, containing one or more sugar moieties. Because of their lipid-soluble aglycone part and water-soluble sugar chains, saponins are amphiphilic molecules and widely used as surface active agents. They exhibit diverse biological activities due to their anti-inflammatory, adjuvant, antifungal, anti-parasitic, antioxidant, anti-obesity, antiviral, diuretic, hemolytic, and neuroprotective properties (Moses et al., 2014). The capability of saponins to form a complex with steroids, including cholesterol, has been used to promote the formation of stable particulates. Immune stimulating complexes (ISCOMs) are 40–60 nm-sized cage-like structures composed of cholesterol, phospholipids, Quillaja saponins, and antigens. ISCOMATRIX differs from ISCOMs in that the antigen is not integrated into the structure during its synthesis (Sun et al., 2009; Duewell et al., 2011). Influenza ISCOM vaccines induced antibody and cytotoxic T-cell response in mice (Sjölander et al., 2001). 

The crude extract of *Astragalus membranaceus* has been used in traditional Chinese medicine to treat the common cold, influenza, chronic diarrhea, oedema, diabetes mellitus, and so forth. It is rich in saponins, flavonoids, polysaccharides, amino acids, and many trace elements (Ma et al., 2002). Astragaloside VII, a triterpenoid saponin isolated from several *Astragalus* species, demonstrated a Th1/Th2-balanced antibody response along with the production of Th1 and Th17 related cytokines (i.e. IL-2, IFN-γ, IL-17A, and TGF-β) and stimulation of splenocytes to proliferate (Bedir et al., 1999; Nalbantsoy et al., 2011; Nalbantsoy et al., 2012; Yakuboğulları et al., 2019). AST-VII, the first tridesmosidic molecule isolated from natural sources, is highly polar and acid-labile, and its bioavailability from the gastrointestinal tract is questionable. Recently, Cano-Sarabia et al. reported a cholesterol-rich double-layer nanovesicle assembly solid in the form of CO2-expanded acetone, which is driven by the presence of a surfactant and is claimed to be more stable than liposomes (Ferrer-Tasies et al., 2013). However, this method needs special equipment and can only be performed under high pressure. 

Thus, herein we attempted to optimize a more straightforward method that could overcome these constraints and facilitate the formation of the saponin-cholesterol complexation by solvent-assisted nanoprecipitation. We developed lipid nanoparticles to deliver AST-VII in order to increase the immunostimulatory effects of AST-VII on seasonal influenza (H3N2) in vitro. Cholesterol, low molecular weight PEG (MW = 300), and AST-VII were used as the main components of the nanocarrier system. Acetone, ethanol, and chloroform were evaluated as primary solvents modulating the physicochemical properties of the resulting nanoparticles. The H3N2 subset was chosen as a viral antigen. The in vitro immunomodulatory effects of AST-VII-integrated PEG300: Chol nanoformulations (LNPs) were investigated by evaluating the cytokine release profiles in human whole blood and also hemolytic activity. These data were later compared with those of AST-VII and QS-21.

## 2. Materials and methods

### 2.1. Materials

RPMI-1640 (Sigma-Aldrich Corp., St. Louis, MO, USA), PMA (Sigma-Aldrich Corp.), ionomycin (Sigma-Aldrich Corp.), penicillin-streptomycin (Biological Industries, Beit Haemek, Israel), fetal bovine serum (Gibco, Thermo Fisher Scientific Inc.,Waltham, MA, USA), IL-17A, IL-2, and IFN-g ELISA kits from eBioscience (Aviscera Bioscience, Inc., Santa Clara, CA, USA) were used in the experiments. A/duck/Hokkaido/5/77 H3N2, provided and given as a gift by from the Avian Influenza OIE /FAO Reference Lab. of Hokkaido University in Japan, was also used. Polyethylene glycol (MW=300) PEG, Cholesterol, PBS (10X), and solvents were purchased from Sigma-Aldrich Corp.. AST-VII was provided by the Bionorm Company, İzmir, Turkey.

### 2.2. Nanocarrier synthesis via the nanoprecipitation method

The nanoprecipitation method was first conducted to evaluate 3 solvents (chloroform, acetone, or ethanol) possessing different polarities for primary solvent and water as a dispersing phase. Cholesterol (20 mg) and AST-VII (2.5 mg) were dispersed in 1 mL of primary solvent (chloroform, acetone, or ethanol) and mixed drop-wise (approximately 50 µL/s each) with preheated PEG300 (5 mg) dissolved in 5 mL Mili-Q water at 70 °C (higher than the Tg of cholesterol) under vigorous mixing. Resulting nanocarriers were cooled down to room temperature and kept in a refrigerator until further use.

### 2.3. Nanocarrier characterization

Hydrodynamic radius and surface ζ-Pot of the resulting nanoformulation, PEG300: Chol-AST-VII were characterized by dynamic light scattering (DLS) using Nanosizer/Zetasizer Nano-ZS ZEN 3600 (Malvern Panalytical Inc., Westborough, MA, USA). Results were presented as the average and standard error calculated using at least 3 values obtained from parallel experiments under the same conditions. The isoelectric point of the LNPs was determined by measuringζ-Pot values at a range of pH values between 2–12 via Nano-ZS ZEN 3600 (Malvern Panalytical Inc.). Surface characteristics were analyzed by Fourier transform infrared spectroscopy (FTIR) (PerkinElmer, Inc., Waltham, MA, USA). All samples were dried on support and measured in the frequency range of 4000 to 450 cm–1 with 4 cm–1 resolution. Particle morphology was evaluated via a JEOL JEM-1400 120kV, transmission electron microscope (TEM). Particle contrast was enhanced by uranyl acetate staining [2% (w/v), 2 min]. AST-VII entrapment efficiency (EE%) of the prepared LNPs were evaluated by an indirect method. Approximately 2 mL of the sample was purified through a Millipore Amicon Ultra-2.0 centrifugal filter unit by centrifugation of the sample at 7000 rpm for 10 min. The supernatant containing the free AST-VII was collected, and samples were immediately analyzed by HPLC. AST-VII was detected following HPLC conditions: Column Thermo C18: 100 × 4.6 mm, 3 µm; gradient 0–1.50 min 70% water, 30% acetonitrile; 1.50–13 min 45% water 55% acetonitrile; 13–15 min 70% water 30% acetonitrile; temperature 35 ºC; injection volume 5 μL; flow rate 0.75 mL/min.

### 2.4. Preparation, isolation, and inactivation of H3N2

Preparation, isolation, and inactivation studies of H3N2 were performed at the Poultry Diseases Diagnosis Laboratory, Veterinary Control, and Research Institute in İzmir, Turkey. H3N2 was propagated on 9 to 10-d-old embryonated SPF (specific pathogen-free) chicken eggs. The allantoic fluids of the eggs at the end of the incubation period were collected, centrifuged at 6000 rpm for 10 min, and then tested for hemagglutinating activity. Serial 10-fold dilutions of allantoic fluid were prepared and injected into embryonated SPF chicken eggs to calculate EID50 by the Spearman-Karber method (Allan et al., 1978). The titers of H3N2 were found to be 1:264. H3N2 was inactivated with 8% of BEI (binary ethyleneimine) (Bahnemann, 1975). The inactivated virus sample was also inoculated to 5 SPF eggs and incubated for 3–4 days in order to confirm inactivation. The allantoic fluid was examined over 3 serial passages in the SPF eggs and tested by HA (hemagglutination assay) to determine a surviving virus.

### 2.5. Peripheral whole-blood cultures

Human whole blood (hWB) and hemolytic activity assays were assessed using heparinized whole blood obtained from healthy volunteers (Ege University, Medico-social Policlinic, İzmir, Turkey). The protocol was approved by the Human Ethics Committee of Ege University, and applied procedures conformed to the Declaration of Helsinki. The subjects were informed about the procedures to be followed and signed the informed consent forms (15-11/19).

AST-VII and QS-21 were dissolved in saline, and all preparations were filtered through a 0.22-μM filter. AST-VII, QS-21, and PEG300: Chol-AST-VII LNPs were added into inactivated H3N2 with simple admixture. Heparinized whole blood from healthy volunteers was diluted 1:10 with RPMI-1640 medium, 100 U/mL of penicillin, 100 mg/mL of streptomycin, and 10% fetal bovine serum. PMA (50 ng/mL) and ionomycin (400 ng/mL) stimulated hWB cultures were treated with AST-VII, QS-21, and PEG300: Chol-AST-VII (1:5:20) at 2 concentrations (3 µg/mL and 6 µg/mL), both in the absence and presence of inactivated H3N2 (4HA) and incubated at 37 ºC in 5% CO2 for 48 h (Yesilada et al., 2005). The cell culture supernatants were collected and stored at –20 ºC for further cytokine analysis.

### 2.6. Measurement of cytokine production

IFN-γ, IL-2, and IL-17A titers in the cell culture supernatants were measured using a commercial ELISA kit (e-Bioscience, Vienna, Austria). The assay was performed according to the manufacturer’s instructions and in triplicate. Spectrophotometric analysis was conducted by measuring the sample absorbance at 450 nm, and cytokine levels were determined from a standard curve. The sensitivity of each kit was 2 pg/mL, 4 pg/mL, and 4 pg/mL for IL-2, IFN-γ, and IL-17A, respectively. 

### 2.7. Hemolytic activity assay

Hemolytic activity assay was performed with human red blood cells (RBCs) following the methodology of Nalbantsoy et al. (2011). Blood samples prepared as aliquots of 7 mL were washed and centrifuged 3 times at 2000×g for 5 min with sterile PBS. The cell suspension was prepared by diluting the pellet to 0.5% w/v in PBS (10×, pH 7.4). Approximately 0.01 mL of the cell suspension was mixed separately with AST-VII, QS-21, and PEG300: Chol-AST-VII LNPs (1:5:20) at varying concentrations (0, 25, 50, and 250 µg/mL) in 0.05 mL saline solution. The mixtures were incubated for 30 min at 37 ºC and centrifuged at 800×g for 10 min. The free hemoglobin content of the cell supernatants was determined by measuring optical density at 412 nm using a UV spectrophotometer. PBS and distilled water were defined as minimum and maximum hemolytic controls. The hemolytic activity (%) was calculated by following formula= (A412 of RBCs treated samples – A412 of PBS treatment)/(A412 of distilled water treatment – A412 of PBS). Each experiment was run in triplicate at each concentration.

### 2.8. Statistical analysis

The data were measured with mean and standard errors, and the statistical significance of differences was examined using the Student t-test, one-way ANOVA, and Tamhane’s T2 as the post hoc test (GraphPad Prism 5.01 and SPSS for Windows). Statistically significance was determined as *P < 0.05, **P ≤ 0.01, and ***P ≤ 0.001.

## 3. Results and discussion

### 3.1. Nanocarrier preparation and characterization

**Figure 1:**
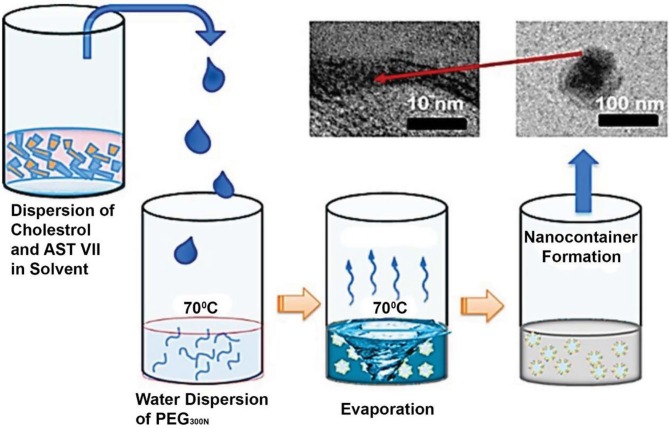
Schematic illustration of the synthesis route applied for the formation of PEG300: Chol-AST-VII LNPs through the nanoprecipitation method in the presence of solvent/water interphase. TEM image of an as-synthesized nanovesicle and magnified image of the nanovesicle membrane.

Cholesterol-rich nanocarriers were prepared through the nanoprecipitation method using 3 solvents with different polarities, boiling points as the primary solvent, and water as a dispersing phase: Acetone as an aprotic polar solvent; ethanol as a protic polar solvent; chloroform as a nonpolar, water-immiscible solvent. As depicted in Figure 1, nanoprecipitation occurred instantaneously upon the addition of the primary solution to the PEG (in water), resulting in a whitish emulsion. After 1 h, under argon air supply, a clear solution was obtained with chloroform, while opaque white solutions were observed when ethanol and acetone were used. 

**Figure 2:**
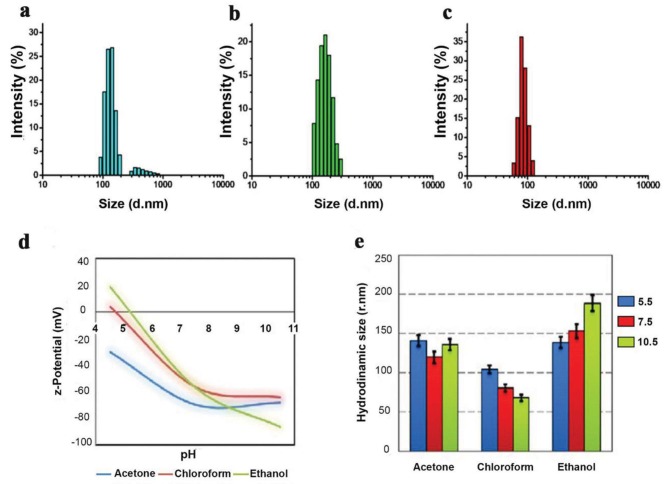
DLS results of PEG300: Chol-AST-VII LNPs. Hydrodynamic particle size distribution by intensity graph of PEG300: Chol-AST-VII LNPs made using (a) acetone; (b) ethanol; and (c) chloroform as a primary solvent for the nanoprecipitation method. Dependence of (d) ζ-Potential and (e) hydrodynamic size of nanocarriers on the pH and primary solvent type.

Hydrodynamic particle size distribution by intensity was measured using the DLS method and depicted in Figure 2. LNPs were prepared in both of the solvents and showed a polydispersity index below 0.5, attributed to a homogenous size distribution. In acetone assisted preparation (Figure 2a), 2 groups of nanoparticle populations appeared: 1 group with a larger Rh around 300–1000 nm and the other with a lower Rh value around 132.9 ± 10.1 nm. In ethanol, the Rh of particles decreased to 169.1 ± 9.8 nm (Figure 2b). In the presence of chloroform, LNPs at 85.3 ± 6.3 nm with narrower size distribution [Polydispersity index (PDI = 0.218] were observed. The resulting LNPs are (Figure 2c) in the same size range as the Quatsomes reported by Ferrer-Tasies. (Ferrer-Tasies et al., 2013). Decreased Rh in the presence of water-immiscible chloroform could be due to solvent displacement, which generates interfacial turbulence and leads to nanovesicle formation (Campardelli et al., 2012). During the displacement, the first aggregates split to form nanodroplets, and once the solvent migrated through the water, they transformed into nanocarriers; when the displacement rate was faster, the nanocarrier size was smaller, which is consistent with results reported here. 

The effect of different primary solvents on the ζ-Pot and the isoelectric point of LNPs are shown in Figure 2d. Contrary to the highly positive ζ-Pot values of cholesterol-rich nanocarriers reported in the literature (Cano-Sarabia et al., 2009), the LNPs prepared here showed a surface with a highly negative ζ-Pot. This pronounced difference may be evidence of the integration of AST-VII and PEG molecules with the arrangement of oxygen-rich surface functional groups approaching the cholesterol-based membrane surface. The ζ-Pot of PEG300: Chol-AST-VII LNPs synthesized via ethanol, and chloroform (–56.9 and –56.3 mV, respectively) was quite similar to each other except for PEG300: Chol-AST-VII LNPs prepared with acetone that had a higher ζ-Pot value (–69.8 mV), indicating that the charge density significantly depends on the solvent. 

**Figure 3:**
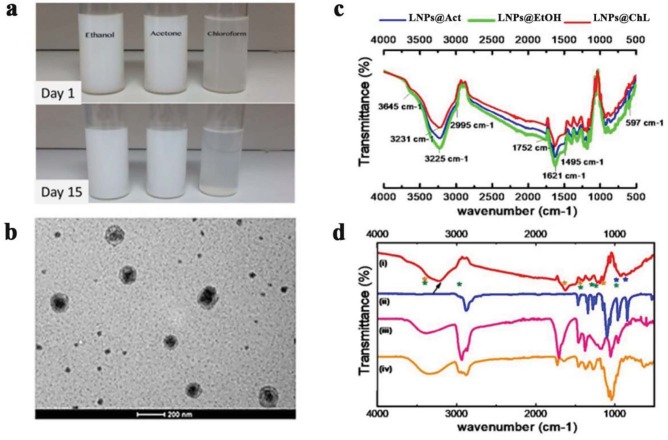
TEM and FTIR analysis of LNPs. (a) images of PEG300: Chol-AST-VII nanocarriers prepared using different primary solvents before and after storage at room temperature over 15 min. No precipitation was observed over time; (b) transmission electron microscope image of PEG300: Chol-AST-VII LNPs made up using chloroform as a primary solvent; (c) FT-IR spectra of AST-VII integrated nanocarriers prepared using chloroform (red line), acetone (blue line), and ethanol (green line), spectra corresponding to (d) PEG300: Chol-AST-VII LNPs prepared in chloroform/water (i) and each component of LNPs: cholesterol (ii), Polyethylene glycol (iii), and AST-VII (iv). The characteristic peaks of each component are illustrated by asterisks (*); the arrow points to the interassociated hydrogen bonds formed by carrier formation.

Figures 2d and 2e showed the average zeta potential (ζ-Pot) and hydrodynamic radius (Rh) of prepared PEG300: Chol-AST-VII LNPs as a function of pH. Similarly, the difference in particle surface chemistry due to the desolvating agent affected their surface response to varying pHs (pH 4–11). The isoelectric point (pI) for each sample was calculated by the polynomial fitting of the data plotted in Figure 2d (ζ-Pot values versus pH). The pI value of nanocarriers prepared in acetone, ethanol, and chloroform was 5.15, 3.12, and 4.71, respectively. As presented in Figure 2e, the Rh of LNPs prepared in acetone as a primary solvent did not show significant pH dependence. The Rh of PEG300: Chol-AST-VII LNPs prepared in chloroform decreased with increased alkalinity. That could be explained by a favored electrostatic repulsion at higher pH values. On the contrary, when LNPs formed in ethanol, particle size increased simultaneously with increasing pH. The arrangement of PEG and AST-VII throughout the cholesterol membrane resulted in a less soluble surface at acidic medium; therefore, at lower pHs, the hydrophobic effect dominates protonation, and nanocarriers shrink as a result (Chen and Du, 2013). As shown in Figure 3a, no flocculation or precipitation was observed in either of the samples after storage at 25 °C for 2 weeks; the samples remained dispersed in the liquid and did not settle out. 

Figure 3b shows the morphology of PEG300: Chol-AST-VII LNPs prepared using the chloroform that has the lowest nanocarrier Rh with narrower size distribution. As indicated in Figures 1 and 2c, PEG300: Chol-AST-VII LNPs displayed a uniformly distributed particle size with semispherical geometry. A membrane with a thickness of 4–5 nm corresponds to a bilayer membrane (Genç et al., 2009; Ferrer-Tasies et al., 2013).

As disclosed in Figure 3b, the Rh of PEG300: Chol-AST-VII LNPs matched the average diameter shown in Figure 2c. Besides this, Figure 3b demonstrates a thick surface layer comprising of a hollow internal region. The surrounding shadows of PEG300: Chol-AST-VII LNPs were made up of uranyl acetate, which was used for a sharper contrast between the nanocarriers and the background.

**Figure 4:**
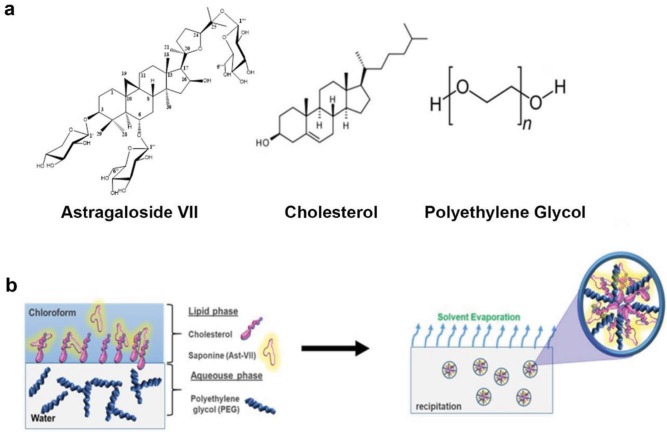
Chemical representation of each component of LNPs. a) AST-VII, cholesterol and polyethylene glycol; b) schematic representation of LNP formation in chloroform/water interphase.

In order to assess the particle surface chemistry, FT-IR analyses of liquid samples were performed. IR spectra of the pristine, AST-VII, and nanocarriers, obtained using different primary solvents, are shown in Figure 3c. As expected, they all showed several characteristic bands of the AST-VII, PEG, and cholesterol (Figure 3d). The broad transmission band centered around 3400 cm–1, was assigned to the hydrogen-bonded hydroxyl groups of both AST-VII and cholesterol, and became less prominent in self-assembled carriers. More importantly, a shoulder band with a stronger absorbance peak appeared at slightly lower frequencies (~3220 cm–1) (Figure 3d). This can be presumed as evidence of the interassociated hydrogen bonds formed between each component (Salim et al., 2013). The increased transmittance in this region also reflects the decreased hydrophilicity of the surface. The reduced intensity of the main bands of AST-VII assigned to alkyl groups (2700–3000 cm–1), together with the disappearance of the C-H bend at 1039 cm–1, demonstrates that AST-VII was embedded through the cholesterol membrane by hydrophobic carbonaceous groups (Figures 3c and 3d). The same IR profiles were present for all the nanocarrier suspensions with changing transmittance regardless of the solvent used (Figure 3c). A proposed nanocarrier configuration with each component of the formulation is represented in Figures 4a and 4b. PEG300: Chol-AST-VII LNPs with the lowest size distribution and opacity were observed when nanocarriers were prepared using chloroform, therefore further in vitro studies were pursued with those carriers. The AST-VII entrapment efficiency of the LNPs was determined as 65 ± 5.3% with the HPLC method.

### 3.2. Evaluation of cytokine response induced by PEG300: Chol-AST-VII LNPs adjuvanted H3N2 vaccines in vitro

A comparative analysis of PEG300: Chol-AST-VII LNPs on human whole blood (hWB) was conducted to screen the cytokine profile (IL-2, IFN-γ and IL-17A) and a possible type of immune response towards Th1/Th2/Th17. 

**Figure 5:**
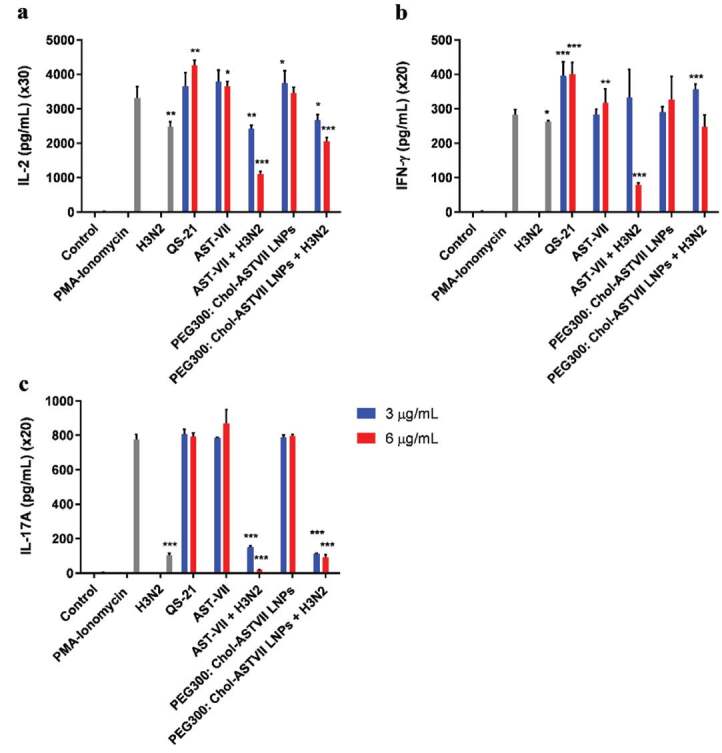
Cytokine profile of adjuvants and adjuvanted H3N2. Cytokine levels of PMA-ionomycin stimulated hWB treated with ASTVII, QS-21, and PEG300: Chol-AST-VII LNPs mixed w/without inactivate H3N2. The cell culture supernatants were analyzed for a) IL-2; b) IFN-γ; and c) IL-17A by ELISA. The significant difference is defined between treated groups and PMA-ionomycin: *P < 0.05, **P < 0.01, ***P < 0.001.

IL-2 is a Th1 type cytokine and does not have direct antiviral activity; however, it has an essential role in the modulation of many immunologic events by stimulating the rapid proliferation of T-cells (Liao et al., 2013). As shown in Figure 5a, IL-2 levels significantly changed when cells were exposed to 6 µg/mL AST-VII (P < 0.05) and QS-21 (P ≤ 0.01). The IL-2 level in the PEG300: Chol-AST-VII LNPs (3 µg/mL) treatment group was 1.12-fold higher than the PMA-ionomycin treatment, which was also higher than the AST-VII (1.14 fold) treatment. When cells were exposed to the H3N2 vaccine mixed with AST-VII at 6 µg/mL (P ≤ 0.001) and PEG300: Chol-AST-VII LNPs at both concentrations [3 µg/mL (P < 0.05) and 6 µg/mL (P ≤ 0.001)], IL-2 levels dropped in comparison to PMA-ionomycin. The decrease was as high as 3.85-fold for AST-VII treated cells, while encapsulation of AST-VII in LNPs backbone decreased this influence to 1.4-fold (3 µg/mL) and 1.77-fold (6 µg/mL), respectively. Inactivated H3N2 (4HA) alone also decreased the IL-2 titers by 1.3-fold (P ≤ 0.01), compared to PMA-ionomycin. These results show that the integration of AST-VII into a nanocontainer reduced the immune-suppressive properties of influenza regarding H3N2 combined with AST-VII alone. There are many reports demonstrating a similar retardation or passivation effect of nanoparticles by a slow-release that could be useful for preventing or controlling the immune-suppressive properties of therapeutic cargo (Higaki et al., 2005).

IFN-γ is produced by Th1 and NK cells and is direct evidence of its antiviral effect (Han and Meydani, 2000). As depicted in Figure 5b, treatment of AST-VII (6 µg/mL; P ≤ 0.01) and QS-21 (3 µg/mL; P ≤ 0.001; 6 µg/mL: P ≤ 0.001) promoted IFN-γ secretion significantly in respect to PMA-ionomycin. PEG300: Chol-AST-VII LNPs alone did not affect IFN-γ levels at the concentrations of 3 and 6 µg/mL, whereas mixing PEG300: Chol-AST-VII LNPs (3 µg/mL) with inactivated H3N2 substantially elevated IFN-γ levels by 1.25-fold (P ≤ 0.001). The increased concentration of PEG300: Chol-AST-VII LNPs to 6 µg/mL decreased the IFN-γ concentration to the levels expressed by AST-VII (6 µg/mL) alone. Depletion of the IFN-γ titers (P ≤ 0.001) occurred in the presence of AST-VII (6 µg/mL), and the inactivated H3N2 mixture was 3.32 times higher than H3N2 alone (P < 0.05). It is already known that cationic cholesterol combined with split inactivated influenza vaccines displayed remarkable adjuvant properties in mice and macaques (Guy, 2007). The aforementioned result might be related to the presence of cholesterol and its ability to produce IFN-γ, but the dominant mechanism for our formulation has yet to be elucidated (Barnier-Quer et al., 2013).

IL-17A is a proinflammatory cytokine produced by Th17 cells, CD8+ T cells, NKT cells, neutrophils, and macrophages and acts as an inflammation mediator (Xu and Cao, 2010). As shown in Figure 5c, no significant augmentation in IL-17A levels was observed even at the highest concentrations of AST-VII, PEG300: Chol-AST-VII LNPs, or QS-21. When the cells were co-treated with AST-VII and PEG300: Chol-AST-VII LNPs at concentrations of 3 and 6 µg/mL and inactivated H3N2, IL-17A titers significantly decreased 5.2-fold, 44-fold, 7-fold, and 8.5-fold, compared to the PMA-ionomycin treated group, respectively (P ≤ 0.001).

**Figure 6:**
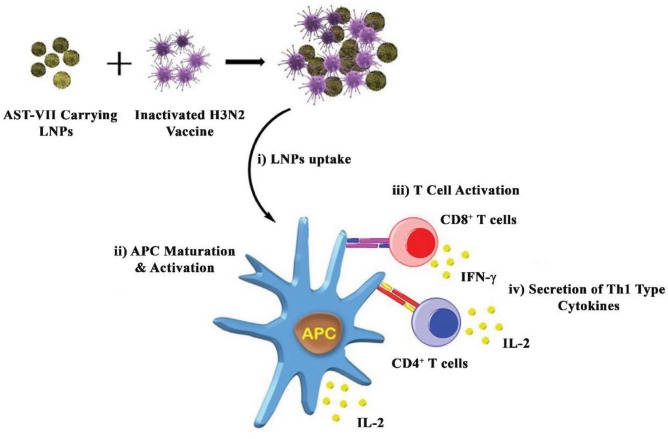
Schematic representation of the possible mechanism of action for PEG300: Chol-AST-VII LNPs. Inactivated H3N2 vaccine combined with AST-VII-carrying LNPs, and this combination was taken by APC (i). After internalization of the adjuvant-antigen combination, APC (immature state) was processed and presented the viral antigens on the MHC molecules (mature state-activated) (ii) to naïve CD8^+^ and CD4^+^ T-cells (iii). Activated T-cells secreted Th1 type cytokines (i.e. IL-2, IFN-g).

PEG300: Chol-AST-VII LNPs revealed a similar response with the LNPs against IVs in the literature that promoted the production of Th1 cytokines, IL-2, and IFN-g but suppressed the IL-17A response compared to PMA-ionomycin. The cationic liposome adjuvant system (CAF01), containing trehalose 6,6’-dibehenate (TDB) and dimethyldioctadecylammonium (DDA), combined with trivalent influenza vaccine (TIV), enhanced influenza-specific serum antibody titers and Th1/Th17 immune response in terms of IL-1b, IL-2, IL-12, IFN-g, TNF-a, and IL-17 secretions in vivo (Davidsen et al., 2005; Rosenkrands et al., 2011) *.* ISCOMATRIX, an LNP containing saponin compound, elicited a Th1/Th2 mixed immune response (IL-2, IFN-g and IL-4) in mice. Also, administration of ISCOMATRIX with influenza promoted high antibody titers and a CD4+ T-cell response (Morelli et al., 2012). Figure 6 illustrates the possible action mechanism of PEG300: Chol-AST-VII LNPs in terms of IL-2 and IFN-g secretion.

We speculate that the influenza virus alters antibody production, T lymphocyte activation, and neutrophil function due to its immunosuppressive properties. Thus, it possibly caused a decrease in phagocytosis, loss of alveolar macrophages, and production/suppression of a variety of cytokines such as IL-10, which is associated with down-regulation of Th1 cytokines such as IL-2 and IFN-γ (McCullers, 2006). Besides, a conserved motif in the fusion peptide of influenza hemagglutinin could be responsible for its immunosuppressive properties, affecting cytokine expression, production of TNF-α, IFN-β, IFN-γ, and inhibition of type I IFN pathway (Marcus et al., 2005). EGR2 (early growth response) is a transcriptional factor and has an essential role in the differentiation of naive T-cells and the regulation of antigen-specific immune responses to the influenza viral infection. T-cells originating from EGR2 conditional knockout mice exhibited a decrease in IFN-γ, TNFα, and IL-2 levels (Du et al., 2014).

Activated T-cells proliferated and differentiated into effector T-cells, which produced cytokines (i.e. IL-2 and IFN-γ) or chemokines, depending on the nature of the antigen.

Our results suggest that LNPs delay or decrease the immunosuppressive properties of the IVs by significantly reversing the inhibitory effect of H3N2 and altering IFN-γ secretion. Therefore, another mechanism related to the influenza immunosuppression could be related to EGR2 signaling, and further studies may exhibit the impact of PEG300: Chol-AST-VII LNPs on this pathway. 

### 3.3. Hemolytic activity

Hemolytic activity was investigated by treating human RBCs with AST-VII, QS-21, and PEG300: Chol-AST-VII LNPs at varying concentrations (25, 50, and 250 µg/mL). As shown in the Table, AST-VII had few or no hemolytic activity, while the same concentrations of QS-21 caused higher hemolysis. AST-VII-carrying LNPs, on the other hand, displayed hemolytic activity only at the highest concentrations (250 µg/mL, 112.10%). PEGylation is a widely used method to overcome the solubility and hemolytic activity problems of drugs (Alayoubi et al., 2013). The bulky PEG head groups on the surface of nanoemulsions prevent interactions with the cell membrane and thus reduce hemolysis (Jumaa et al., 1999). An excess amount of cholesterol in LNPs could prevent the interaction of saponins with cell membrane cholesterols and eventually reduce the hemolysis.

**Table:** Hemolytic activity of AST-VII, QS-21, and PEG300: Chol-AST-VII LNPs in different concentrations. Minimal and maximum hemolytic control was defined as PBS and distilled water, respectively. Significance level was defined as *P < 0.05, ***P ≤ 0.001.

Groups	Absorbance value	Hemolytic activity (%)
PBS	0.1104 ± 0.0010	0.00 ± 0.1880
Distilled water	0.6387 ± 0.0005	99.9937 ± 0.0892
AST-VII (250 µg/mL)	0.1217 ± 0.0012	2.1326 ± 0.2361*
AST-VII (50 µg/mL)	0.1210 ± 0.0022	2.0064 ± 0.4089
AST-VII (25 µg/mL)	0.0873 ± 0.0021	–4.3662 ± 0.3889
QS-21 (250 µg/mL)	0.8737 ± 0.0058	144.4760 ± 1.0965***
QS-21 (50 µg/mL)	0.7857 ± 0.0039	127.8188 ± 0.7304***
QS-21 (25 µg/mL)	0.5147 ± 0.0104	76.5222 ± 1.9691*
PEG300: Chol-AST-VII LNPs (250 µg/mL)	0.7027 ± 0.0050	112.1080 ± 0.9443***
PEG300: Chol-AST-VII LNPs (50 µg/mL)	0.0881 ± 0.0063	–4.2148 ± 1.1966
PEG300: Chol-AST-VII LNPs (25 µg/mL)	0.0719 ± 0.0096	–7.2812 ± 1.8134

In conclusion, this study attempted to develop a new adjuvant delivery system that is effective towards cell-mediated immune response. Self-assembled nanocarriers were designed, taking into account the complexation capacities of saponins with cholesterol. Co-treatment of H3N2 with AST-VII-carrying LNPs induced a higher Th1 response by increasing IL-2 and IFN-γ levels and suppressing IL-17A. Moreover, a minimized hemolysis profile was obtained by PEG300: Chol-AST-VII LNPs compared to QS-21. The data reported here suggest the potential of LNPs to deliver AST-VII and activate Th1-mediated cytokines against H3N2.

## Informed consent

The protocol for human whole blood assay was approved by the Human Ethics Committee of Ege University and applied procedures conformed to the Declaration of Helsinki. The subjects were informed about the procedures to be followed and signed the informed consent forms accordingly (15-11/19).
